# Comparative Analysis of Gender-Based Differences in Behavioral Mastery, Goals, and Characteristics in Autistic Individuals: An Applied Behavior Analysis Study

**DOI:** 10.7759/cureus.62427

**Published:** 2024-06-15

**Authors:** Tami Peterson, Jessica Dodson, Robert Sherwin, Frederick Strale

**Affiliations:** 1 Hyperbaric Oxygen Therapy, The Oxford Center, Brighton, USA; 2 Applied Behavior Analysis, The Oxford Center, Brighton, USA; 3 Hyperbaric Oxygen Therapy, Wayne State University School of Medicine, Detroit, USA; 4 Biostatistics, The Oxford Center, Brighton, USA

**Keywords:** gender comparison, gender-differences, behavioral target mastery, autism spectrum disorder (asd), intensity and duration, open targets, maintenance failure, applied behavioral analysis (aba)

## Abstract

Introduction

It is widely recognized that the prevalence and diagnosis of autism spectrum disorder (ASD) are more common in males than in females. Despite this, there is a significant gap in the body of autism research that investigates gender differences for treatment effects of applied behavior analysis (ABA) across a variety of measured variables. This research aims to comprehensively evaluate gender distinctions concerning target behavioral objectives, goals, and deficit variables.

Materials and methods

This study analyzed retrospective data from 100 participants, including 89 juveniles and four adults, with seven cases lacking age documentation, who underwent a three-month ABA program from March 19 to June 11, 2023. The ABA program included various methodologies such as functional analysis, discrete trial training, mass trials, and naturalistic training. Data on outcome measures, including target behavioral proficiency, age, average trials to proficiency, average teaching days to proficiency, open behavioral objectives, and target trends, were collected using the “Catalyst” software (Catalyst Software Corporation, New York, NY). Participant demographics were summarized using statistical analyses for categorical (gender and race/ethnicity) and continuous variables (percentage of mastered behavioral objectives, age, average trials, average teaching days, open objectives, percentage of failed objectives during maintenance, percentage of objectives with upward, downward, and flat trends). These statistics included mean, standard deviation, median, and range and were analyzed inferentially using nine separate two-sample independent t-tests and corresponding effect sizes using Cohen's d.

Results

There were no statistically significant disparities based on gender (p > 0.05) across all nine variables examined: Percentage of Targets Mastered, Age, Average Trials to Mastery, Average Teaching Days to Mastery, Open Targets, Percentage of Targets Failed in Maintenance, Percentage of Targets Trending Up, Percentage of Targets Trending Down, and Percentage of Targets Trending Flat, and wide confidence intervals were detected.

Conclusions

Non-significant gender differences in response to ABA treatments regarding these nine behavioral goals, mastery, and deficit variables may be relevant. They suggest that ABA treatments could be equally beneficial for both male and female autistic individuals. These results should be interpreted cautiously. The general pattern observed, characterized by broad confidence intervals, carries a degree of statistical uncertainty, which may suggest substantial gender differences. These results might question the prevailing beliefs about the variation in treatment response based on gender. This could profoundly impact clinical practices, implying that healthcare professionals should not favor one gender over another when suggesting ABA therapies. Instead, the treatment advice should be tailored to each child's unique requirements and traits, regardless of gender. The investigators expect these results to encourage additional research in this field. Comprehending the elements that affect treatment response is vital for improving treatment results and customizing care.

## Introduction

Autism spectrum disorder (ASD) is more commonly diagnosed in boys than girls. As per the data from the Centers for Disease Control and Prevention (CDC), in March 2023, about 4% of eight-year-old boys and 1% of eight-year-old girls were diagnosed with autism. Recent studies suggest that autism in females might be underrecognized and incorrectly diagnosed [1].

The manifestation of autism varies in presentation between males and females. Females with autism often exhibit a stronger desire for social interaction, engage in more camouflaging behaviors, and are more likely to adhere to gender norms than males [1]. Their interests, which align more with societal expectations, namely animals, art, celebrities, or literature, can sometimes be misconstrued as intense hobbies rather than traits of autism [1]. 

The differences in how autism presents in different genders could contribute to the underdiagnosis and misdiagnosis of autism in females [2]. Instead of an autism diagnosis, females are often diagnosed with anxiety, mood disorders, learning disorders, and eating disorders [1].

Recent research in brain structure and function indicates that girls with ASD may have more genetic mutations than boys with ASD. Additionally, there are differences in brain structure and function between girls and boys with ASD [3]. 

Current research on gender diversity reveals individuals with autism have a higher prevalence of gender diversity than neurotypical individuals. This has led to a gradual shift in autism research to include a broader representation of gender diversity [4]. 

Gender differences with autism are a complex and rapidly evolving field of study, and our understanding continues to expand with ongoing research. Everyone with autism is unique, and these general trends may not apply to everyone.

Peterson et al. explored the efficacy of ABA interventions for individuals with ASD through repeated measures designs, revealing notable positive and statistically significant advancements in assessed cumulative target behaviors over time [5-8]. This research contributes to the existing body of evidence supporting the effectiveness of ABA interventions for both children and adults with ASD.

Differential efficacy of applied behavior analysis based on gender 

Research on the gender-specific impacts of ABA on target behaviors in individuals with ASD is somewhat scarce. ASD manifests in diverse ways, with varying symptoms, severity, and co-occurring conditions. This diversity can affect how an individual responds to ABA therapy [9]. It is essential to consider cultural and neurodiversity factors, as they can significantly shape research, practice, and discussions among various stakeholder groups [10]. The broad spectrum of procedures in ABA analyzes the elements that contribute to its effectiveness, which is quite intricate [11-13].

A study by Cariveau et al. [3] examined gender differences in core symptoms, associated features, and treatment response in a sample of 682 youth (585 males, 97 females) with ASD. The participants, aged between three and 17 years (average age = 7.4 years), were part of six federally funded, multisite, randomized clinical trials. The researchers found no significant gender differences in the clinical characteristics of youth with ASD and their response to treatment [13]. 

Another study by Tiura and colleagues found that gender predicted behavioral growth rates in response to ABA interventions [12-14]. Male subjects showed quicker improvement in adaptive behavior and physical development. However, the study had a significant gender imbalance with 27 male and eight female participants (ratio 3.4:1). This small sample size of female participants may have resulted in limited variability. The researchers also noted that other studies did not find gender to be a predictor of treatment outcomes [15-17].

Khasawneh conducted an experimental study with 100 autistic individuals. The experimental group consisted of 50 individuals (30 males and 20 females) with an average age of 6.8 years (SD = 1.2). The control group also included 50 individuals with a slightly younger average age of 6.5 years (SD = 1.5), including 35 males and 15 females. Khasawneh found that male participants had significantly lower scores on the Stereotyped Behavior Scale (SBS) than female participants (p=0.039). This result suggested that evidence-based ABA treatments were more effective in reducing stereotypical behaviors among male participants than female participants [16].

Studies assessing the efficacy of intensity, duration, and age on target mastery

Numerous studies have investigated the influence of factors such as age [18-21], intensity of treatment [18,21-26], and duration of treatment [21,27,28] on the outcomes of ABA therapy in individuals diagnosed with autism [28]. Linstead et al. have emphasized the necessity for more comprehensive research regarding the impact of treatment duration. They cited Granpeesheh et al., who proposed the utilization of mastered learning objectives as a dependent variable in treatment studies to assess short-term outcomes, which are typically overlooked by standardized diagnostic assessment scales. This approach offers a socially relevant and broad evaluation of treatment progress. Linstead et al. stressed the significance of treatment-specific assessments of mastered learning objectives to assess short-term advancements or setbacks. They also emphasized the pivotal role of treatment duration and intensity as key predictors of mastering behavioral targets [17,22,28].

Moreover, employing large sample size (Large N) designs to predict future learning rates and treatment-specific variables can provide clinicians, educators, policymakers, and parents with insights into the potential response of individuals with ASD to ABA treatment. Further investigation into the relationship between treatment duration and skill acquisition could furnish valuable information to clinicians and parents regarding potential treatment outcomes, thus, mitigating dropout rates and fostering increased parental engagement in treatment [18].

In autism research, there is a notable scarcity of studies examining gender differences across various measured variables, encompassing age, treatment intensity, and duration, as well as the average number of trials and teaching days required to achieve behavioral mastery. Additional variables of interest include the percentage of targets where maintenance was not achieved, termed as behavioral maintenance failure, and open behavioral targets, referring to objectives that individuals have yet to master.

Furthermore, there is a lack of research focusing on behavioral trend variables. These encompass the percentage of targets trending upward, indicating improvement; the percentage trending downward, indicating regression; and the percentage of targets that remain flat, indicating no significant change.

The scarcity of such gender-focused studies underscores the need for more comprehensive research in this area. This research could provide valuable insights into the differential impacts of various factors on the effectiveness of ABA treatment among different genders. It could also contribute to developing more personalized and effective treatment strategies for individuals with autism.

Objectives 

This study aims to assess the efficacy of ABA treatment in individuals who have been diagnosed with ASD and to investigate any gender-based variations in the response to treatment. The variables under investigation encompass age, intensity, and duration of therapy, evaluated through both the average number of trials required to attain behavioral mastery and the average number of teaching days needed for the same. Furthermore, the study will analyze the frequency of behavioral maintenance failure, indicated by the percentage of targets where maintenance was not achieved, along with the quantity of open behavioral targets, denoting objectives not yet mastered by the individual. Additionally, behavioral trend variables will be examined, including the percentage of targets displaying upward, downward, or stagnant trends. All with a focus on possible gender differences.

## Materials and methods

Research setting and subjects

This study involved a retrospective examination of a cohort comprising 100 individuals diagnosed with ABA, including 89 children and four adults. Seven instances of age data were missing. The selection criteria for participants in this study were specific individuals diagnosed with autism who received ABA treatment at The Oxford Centers (TOCs) in Brighton and Troy, Michigan, between March 19, 2023 and June 11, 2023 (TOC specializes in a comprehensive approach to ABA, incorporating discrete trial training, mass trials, and naturalistic environment training modalities). These individuals underwent a three-month ABA treatment regimen, incorporating functional analysis, discrete trial training, mass trials, and naturalistic training. Data relative to target behavioral mastery, age, average trials to mastery, average teaching days to mastery, open behavioral targets, percentage of targets failed in maintenance, percentage of targets trending up, percentage of targets trending down, and percentage of targets trending flat was collected.

Before training, each participant received a personalized treatment plan tailored to their needs and objectives, developed by one of eight board-certified behavioral analysts (BCBAs). Participants were then assigned to one of 83 behavioral technicians, with a team comprising three to five technicians working with each participant over three months. Suitable materials were selected and arranged in designated rooms for individual discrete trial training, mass trials, or naturalistic settings, facilitating interaction and engagement in real-world situations. Each behavioral technician was allocated to a different participant daily, delivering an average of four to seven hours of treatment per day, with a minimum of 25 hours per week.

Teams of behavioral technicians collected specific behavioral and skill data, focusing on antecedents, behaviors, and consequences. They monitored progress and gradually reduced prompts and reinforcements as participants achieved skills with 80% accuracy. They also tracked whether participants were generalizing and maintaining acquired skills. The data were entered into a handheld “Catalyst” database and regularly updated into a central database.

Operational definition of variables and data gathering

Throughout this study, the main outcome variables evaluated over three months included the proportion of mastered behavioral targets, age (in years), average trials needed to achieve behavioral mastery (a gauge of treatment intensity and duration), average teaching days to behavioral mastery (another measure reflecting treatment intensity and duration), open behavioral targets, the percentage of behavioral targets failing in maintenance, the percentage of behavioral targets trending upward, the percentage trending downward, and the percentage maintaining a steady course.

The percentage of mastered behavioral targets assessed an individual's progress toward learning objectives or skills. Mastery was defined as correctly performing a task or skill with 80% accuracy, as outlined by the BCBA's criteria. Age in years represented the individual's chronological age calculated based on a standard calendar year. Average trials to behavioral mastery, indicating treatment intensity and duration, reflected the number of behavioral responses required to reach a predetermined level of performance set by the BCBA for a specific skill or behavior. The average teaching days to mastery, another indicator of treatment intensity and duration, denoted the duration from introducing a target to its mastery. Participants received ABA treatment five days a week, with tailored protocols for each individual.

Open behavioral targets referred to behaviors currently undergoing instruction and learning but not yet mastered. The percentage of behavioral targets failing in maintenance indicated behaviors not sustained over time, suggesting a lapse in demonstrating or retaining previously acquired skills or behaviors once the ABA antecedent was removed. The percentage of targets trending upward represented the proportion of behavioral targets showing increased desired responses over time, indicating progress toward mastering a specific behavior or skill. Conversely, the percentage trending downwards indicated behavioral targets showing decreased desired responses over time, potentially signaling difficulty with a particular behavior or skill or necessitating adjustments to intervention strategies.

Percent of targets trending flat denotes the percentage of behavioral targets showing no significant change in responses over time. A flat trend could indicate that the individual’s performance on a particular behavior or skill has plateaued. These variables are crucial for tracking progress and adjusting treatment plans as necessary. They provide valuable insights into how an individual is responding to ABA treatment.

The ABA data collection software known as “Catalyst” (Catalyst Software Corporation, New York, NY) was employed to produce automated progress reports regarding outcome data for discrete trial teaching objectives, utilizing frequency and rate data. Mastery criteria for these target behaviors were delineated based on the percentage of behavioral trials, the minimum number of behavioral trials, and the involvement of therapists surpassing the 80% criterion. Graphs within Catalyst were tailored to monitor advancements or setbacks in targeted behaviors. When the criteria were met, Catalyst automatically identified mastered target behaviors. 

Descriptive and inferential statistics

All statistical analyses were performed using IBM SPSS Statistics for Windows, Version 29.0 (IBM Corp., Armonk, NY, USA) [29]. A nominal α level of 0.05 was set. If the p-value is < 0.05, the null hypothesis would be rejected, indicating statistical significance. Descriptive statistics for demographic data were computed and presented, including any instances of missing data. Summary statistics were calculated for both categorical variables (gender and race/ethnicity) and continuous variables (age, percentage of mastered behavioral targets, average trials to mastery, average teaching days to mastery, open behavioral targets, and percentage of failed behavioral targets in maintenance). These summary statistics encompassed the mean, standard deviation, median, and range.

Inferential statistics comprised nine separate two-independent-sample t-tests with gender serving as the grouping variable and included 95% confidence intervals (CIs) with effect sizes as measured by Cohen's d. These tests were conducted to examine differences in the following variables: behavioral targets mastered, age, average trials to mastery, average teaching days to mastery, open behavioral targets, percentage of targets failed in maintenance, percentage of targets trending upward, percentage of targets trending downward, and percentage of targets maintaining a steady course.

Ethics committee

This retrospective research study was conducted using data obtained from a thorough review of clinical records. The study underwent review by the Western Institutional Review Board-Copernicus Group (WCG® IRB) and was granted an exemption (approval number: 1-1703366-1). The authors confirm that the analysis adhered to the ethical guidelines outlined in the 1964 Declaration of Helsinki, along with its subsequent revisions, or equivalent ethical standards.

## Results

Descriptive statistical demographics

In this study, a sample of 100 individuals diagnosed with autism was analyzed. Their ages ranged from one to 73 years, with an average age of 8.88 years and a standard deviation of 8.05 years. The median age was seven years. Seven instances were identified where age data was not available. Most of the sample was male, accounting for 74 individuals (74%). There were 25 females (25%). One data point for gender was missing. Regarding ethnicity, the predominant group was white (72%), followed by Asians (12%), American Indian/Alaska Natives (5%), and Hispanics (4%). Seven individuals did not specify their ethnicity.

When classified by age, 18 children (18%) fell into the one to four-year category, 39 children (39%) were in the five to eight-year category, 20 children (20%) were in the nine to 12-year category, and 12 children (12%) were in the 13-16-year category. Four individuals (4%) were aged between 17 and 73 years. There were seven instances where data on age categories were missing. Four subjects were older than 17, precisely 18, 20, 25, and 73 (Table 1).

**Table 1:** Descriptive demographic statistics SD = Standard deviation

Age		
n	93	
Missing	7	
Mean	8.88	
Median	7	
SD	8.05	
Minimum	1	
Maximum	73	
Age Category	n	%
1-4	18	18
5-8	39	39
9-12	20	20
13-16	12	12
17-73	4	4
Missing	7	7
Total	100	100
Gender	n	%
Male	74	74
Female	25	25
Missing	1	1
Total	100	100
Race	n	%
White	72	72
Asian	12	12
Unspecified	7	7
American Indian or Alaska Native	5	5
Hispanic	4	4
Missing	0	0
Total	100	100

Inferential statistics

All nine variables examined did not reveal statistically significant differences between genders (p > 0.05), and their CIs were wide. Cohen's d revealed small effect sizes and wide CIs. For the “Percent of Targets Mastered,” there was no significant difference in means between males (M = 54.2039 (±27.6571)) and females ((M = 57.6628 (±24.6384)), (t(97) = -0.555, p = 0.580, mean difference = -3.4589, CI for mean difference = -15.829, 8.911, d = -0.128, CI for effect size = -0.582, 0.326).

Similarly, for “Age,” there was no significant difference in means between males (M = 9.1149 (±8.8521)) and females (M = 8.125 (±5.1178)) (t(91) = 0.532, p = 0.596, mean difference = 1.0199, CI for mean difference = -2.785, 4.825, d = 0.126, CI for effect size = -0.339, 0.591).

Additionally, for “Average Trials to Mastery,” there was no significant difference in means between males (M = 116.8072 (±81.6977)) and females (M = 123.914 (±97.1463)), (t(97) = -0.358, p = 0.721, mean difference = -7.1068, CI for mean difference = -46.491, 32.277, d = -0.083, CI for effect size = -0.536, 0.371).

Similar patterns were observed for “Average Teaching Days to Mastery,” “Open Targets,” “Percent of Targets Failed in Maintenance,” “Percent of Targets Trending Up,” “Percent of Targets Trending Down,” and “Percent of Targets Trending Flat.” As indicated, none of the nine variables demonstrated statistically significant gender differences (p > 0.05), and the CIs remained wide. Cohen's d suggested small effect sizes with broad CIs. Refer to Table 2 for detailed results.

**Table 2:** Two-independent sample t-test for gender differences SD=Standard Deviation, CI=Confidence Interval

Outcome variable	t	Degrees of freedom	P-value (Two-Tailed)	Male, mean	Male, SD	Female, Mean	Female, SD	Mean difference	95% CI for mean difference		Effect size for Cohen's d	95% CI for Cohen's d	
									Lower	Upper		Lower	Upper
Percent of Targets Mastered	-0.555	97	0.580	54.2039	27.6571	57.6628	24.6384	-3.4589	-15.829	8.911	-0.128	-0.582	0.326
Age	0.532	91	0.596	9.1149	8.8521	8.1250	5.1778	1.0199	-2.785	4.825	0.126	-0.339	0.591
Average Trials to Mastery	-0.358	97	0.721	116.8072	81.6997	123.914	97.1463	-7.1068	-46.491	32.277	-0.083	-0.536	0.371
Average Teaching Days to Mastery	0.516	97	0.607	17.3108	10.4528	16.096	9.2662	1.2148	-3.455	5.8885	0.119	-0.335	0.573
Open targets	1.057	97	0.293	32.6757	19.1168	28.280	13.9777	4.3956	-3.861	12.652	0.244	-0.211	0.699
Percent of Targets Failed in Maintenance	-1.112	97	0.269	4.7214	7.1833	6.5876	7.4802	-1.8662	-5.196	1.466	-0.257	-0.711	0.198
Percent of Targets Trending Up	0.636	97	0.526	12.0669	11.5456	10.4904	7.6259	1.5765	-3.341	6.493	0.147	-0.307	0.601
Percent of Targets Trending Down	-0.473	97	0.637	9.0974	7.9592	10.1428	13.2538	-1.0453	-5.428	3.338	-0.110	-0.563	0.344
Percent of Targets Trending Flat	-0.542	97	0.589	4.6366	8.7124	5.7016	7.7857	-1.0649	-4.964	2.834	-0.125	-0.579	0.329

## Discussion

The present study aimed to evaluate the effectiveness of ABA treatment while focusing on potential gender differences. By examining a comprehensive set of variables, including age, treatment intensity and duration, behavioral maintenance, and behavioral trend variables, this research sought to provide insights into how individuals respond to ABA interventions over three months.

The findings of this study revealed a notable lack of statistically significant gender differences across the measured variables. Despite the importance of gender considerations in treatment outcomes, our results suggest that, within the scope of this study, gender does not play a significant role in predicting response to ABA treatment. This non-significant finding underscores the importance of individualized treatment approaches that address specific behavioral needs irrespective of gender.

The observed trend, however, which is marked by wide-ranging CIs, introduces a level of statistical ambiguity. This uncertainty could potentially indicate significant differences between genders. To elaborate, the broad CIs imply that our statistics could take a wide range of possible values. This wide range, in turn, leads to uncertainty in our statistical analysis. This uncertainty might hint at considerable differences between genders. An ongoing investigation would be needed to confirm this hypothesis. Statistical uncertainty does not necessarily invalidate the study’s findings but highlights areas where more data or refined methodologies might benefit. In this case, it could point investigators toward a fruitful avenue of research into gender differences as it relates to various measured behavioral skill variables relative to the impacts of treatment.

A considerable body of research has explored how variables such as the age of the child [18-21], the intensity of the therapy [18-26], and the length of the treatment period [21-27] can impact the results of ABA therapy in individuals with autism. However, few studies have investigated gender-specific treatment effects of ABA on behaviors in ASD individuals.

ASD is a complex condition that presents itself in many ways, characterized by a range of symptoms, varying degrees of severity, and other concurrent conditions. This complexity and diversity can influence how a person reacts to ABA therapy.

It is crucial to consider factors related to cultural and neurodiversity, as these elements can significantly impact research methodologies, practical applications, and dialogues among different stakeholder groups. The array of procedures within ABA aims to dissect and understand the components contributing to its effectiveness. This analysis is quite sophisticated and intricate, reflecting the complexity of human behavior and the challenges of modifying it in a therapeutic context [11,12].

In essence, while there is a scarcity of research focusing on the gender-specific impacts of ABA therapy on individuals with ASD, the existing studies highlight the complexity of ASD and the intricate nature of ABA therapy. The diversity in ASD manifestations and the influence of cultural and neurodiversity factors underscore the need for a nuanced approach in both research and practice. The broad spectrum of procedures in ABA therapy further emphasizes the intricate nature of this field and the detailed analysis required to understand its effectiveness.

One pivotal aspect of our study centered on assessing behavioral mastery over three months, using mastery criteria set at 80% accuracy for task completion. Intriguingly, gender did not emerge as a significant factor shaping mastery rates, indicating a parallel response to ABA interventions among male and female participants regarding skill acquisition. This observation holds broader implications for the universality of ABA principles in fostering skill acquisition, shedding light on the need for future research to focus deeper on gender-neutral determinants of treatment effectiveness.

This study scrutinized treatment intensity and duration, gauged by the average number of trials and teaching days requisite for achieving behavioral mastery. Despite previous suggestions of gender-based disparities in response to treatment intensity [12-14], our findings did not corroborate such distinctions. Both genders showcased comparable rates of progress and achievement, underscoring the robustness of ABA principles in facilitating skill acquisition [13-16]. Nevertheless, the existence of broad CIs warrants caution in drawing definitive conclusions, necessitating further exploration into nuanced determinants of treatment efficacy.

Analyzing behavioral trend variables was another critical aspect of this study, encompassing upward, downward, and flat trends in response to treatment. While gender-specific disparities in behavioral trends have been hypothesized anecdotally, our study reported no significant differences, signifying the equitable effectiveness of ABA treatment in engendering positive behavioral changes over time for both genders. This underscores the importance of adopting gender-neutral perspectives in elucidating the mechanisms underpinning treatment outcomes and the imperative for future research to unravel the intricate interplay between gender and treatment responses.

This study’s examination of behavioral maintenance failure rates and the number of open behavioral targets revealed no discernible gender differences, implying analogous levels of skill retention and engagement with ongoing intervention targets across genders. Again, wide CIs underscore the necessity for cautious interpretation and prompt further investigation into potential moderating factors shaping treatment outcomes.

These results contribute substantially to elucidating the efficacy of ABA treatment across genders and underscore the imperative of tailoring interventions based on individual needs rather than relying solely on gender considerations. Future research endeavors should strive to unravel the nuanced interactions between gender and treatment outcomes, identifying additional moderators that may influence response to ABA interventions. By embracing a nuanced and inclusive approach to treatment planning and implementation, practitioners can ensure the delivery of effective and personalized interventions for all individuals, transcending gender-specific considerations.

Limitations

This research has certain limitations that need to be emphasized. In terms of sample size and power, wide CIs suggest that the study may have had a relatively small sample size. With a smaller sample size, the study may lack the statistical power to detect subtle but meaningful differences between genders. In future studies, larger samples should be utilized to increase statistical power, thus increasing the likelihood of finding statistically significant differences and reducing the likelihood of a Type II error. As a result, even if there are actual gender differences, they may not be detected due to insufficient statistical power. Furthermore, studies with small sample sizes or wide CIs may struggle with generalizability. The findings may only apply to the specific sample studied and may not accurately reflect the broader population. Therefore, caution should be exercised when extrapolating these findings to other populations. Also, a non-random sample was used for this study; thus, there is no ability to generalize beyond this sample to any larger ASD population. This snapshot study covers three months, and assessing these research subjects over a longer time longitudinally will be informative.

This study’s data also contained considerable variability within gender groups. Gender is a complex construct, and there can be considerable variability within gender groups. Individual differences, cultural influences, and socialization experiences can all impact behavior and response to treatment. Wide CIs may indicate substantial variability within gender groups, making it challenging to draw definitive conclusions about gender differences. Wide CIs may also indicate measurement error or variability in the measurement tools used to assess variables such as age, treatment intensity and duration, and behavioral trends. Measurement error in the form of low interobserver reliability can introduce noise into the data, reducing the precision of estimates and widening CIs.

While the study may not have found statistically significant main effects of gender, there could still be interaction effects between gender and other variables that were not fully explored. For example, gender differences in response to treatment may vary depending on different factors such as age, severity of symptoms, or comorbid conditions. Failure to detect these interaction effects could lead to an incomplete understanding of gender's role in treatment outcomes. Also, comorbidities are common in individuals with autism and may be potential confounders along with other possible confounders. The diversity within ASD suggests the possibility of symptom overlap between ASD and coexisting conditions. Furthermore, the researchers in this study lacked data regarding the various comorbidities prevalent in individuals with autism.

Studies finding statistically significant results are more likely to be published than those with nonsignificant findings. This publication bias can skew the literature, leading to overrepresenting studies reporting significant gender differences and potentially creating a false impression of gender-related effects.

Considering these limitations, future research should replicate and extend these findings using larger, more diverse samples and robust statistical methods. Additionally, qualitative research methods may provide insights into the lived experiences and perspectives of individuals receiving ABA therapy, helping to contextualize quantitative findings and uncover potential gender-related factors influencing treatment outcomes.

## Conclusions

The study found no significant differences between males and females in response to ABA treatments concerning nine specific behavioral objectives, mastery, and characteristics variables. This finding is of considerable interest as it suggests that ABA treatments could be equally effective for autistic individuals of both genders. For clinicians, it implies that the same ABA treatment protocols apply to all individuals with ASD, regardless of gender. This can simplify the treatment process and ensure everyone receives the same high-quality care. It also eliminates any potential bias in treatment delivery based on gender. Gender-neutral interventions can help policymakers ensure equal access to ABA treatments for all individuals with ASD. Policies can be developed and implemented to mandate that ABA treatments be provided without gender discrimination. This can lead to more equitable health outcomes and contribute to the broader goal of health equity. Gender-neutral interventions can improve access to ABA treatments, ensure equal care for all individuals with ASD, and contribute to better overall health outcomes. However, these results should be approached with caution.

The overall pattern observed in the study, characterized by wide CIs, introduces a level of statistical uncertainty. This uncertainty could potentially indicate significant differences between genders. These findings could challenge the existing beliefs about variations in treatment responses based on gender. If validated, they could have a profound impact on clinical practices. They imply that healthcare professionals should not show a preference for one gender over another when recommending ABA therapies. The treatment advice should be individualized, considering each child’s unique needs and characteristics, regardless of gender. This approach ensures that the treatment is tailored to the specific requirements of each child, thereby maximizing its effectiveness. The researchers anticipate that these findings will stimulate further research in this area. Understanding treatment response factors is crucial for enhancing treatment outcomes and personalizing care. By identifying these factors, healthcare professionals can better tailor treatments to each patient's needs, improving their quality of life. This is the ultimate goal of personalized medicine, and this study takes a significant step toward achieving it.
